# Musculoskeletal Complications of Celiac Disease: A Case-Based Review

**DOI:** 10.31138/mjr.34.1.86

**Published:** 2023-03-31

**Authors:** Gerasimos Evangelatos, Konstantina Kouna, Alexios Iliopoulos, George E. Fragoulis

**Affiliations:** 1 First Department of Propaedeutic Internal Medicine, National and Kapodistrian University of Athens, Athens, Greece,; 2 Rheumatology Department, 417 Army Share Fund Hospital (NIMTS), Athens, Greece

**Keywords:** celiac disease, osteoporosis, osteomalacia, arthralgia, back pain

## Abstract

Primary diagnosis of celiac disease (CD) in rheumatology department is not common in daily clinical practice, due to the fact that diarrhoea is usually the dominant symptom. Extra-intestinal manifestations, such as arthralgia, myalgia, osteomalacia, and osteoporosis are not rare in these patients. We present a case of a 66-year-old man, who came to the outpatient rheumatology clinic, complaining of back and knee pain. Osteopenia was observed in plain radiographs, whereas extensive laboratory testing revealed celiac disease, vitamin D deficiency, and extremely low bone mineral density (BMD) due to osteomalacia. Gluten-free diet (GFD) initiation and administration of vitamin D and calcium supplements resulted in significant symptom and BMD improvement over 6 months. A significant proportion of CD patients might present with arthralgia, arthritis, back pain, myalgia, or bone pain. Importantly, up to 75% of patients might have reduced BMD, due to osteoporosis or osteomalacia, while they also carry a significant risk for fracture. However, the introduction of GFD and calcium/vitamin D supplementation significantly ameliorates symptoms and BMD in most cases. Increased awareness of CD’s musculoskeletal manifestations by rheumatologists is important for early recognition and management of this condition and its complications.

## INTRODUCTION

Celiac disease (CD) is an autoimmune inflammatory disease that is triggered by exposure to gluten in genetically sensitive individuals.^1^ The global prevalence is estimated to be 0.5–1%, although it is might be underestimated, due to the wide variety of CD’s manifestations.^1^ Diarrhoea, constipation, abdomen pain, and weight loss are the most common symptoms; however, several extra-intestinal manifestations have reported in about 62% of the patients.^1,2^ Metabolic bone disease is not uncommon and could be present as osteomalacia, osteopenia, or osteoporosis.^3,4^ Moreover, some CD patients might experience myalgia, back pain, arthralgia or frank arthritis.^3,5^ An association between CD and other autoimmune diseases, like autoimmune thyroiditis and type I diabetes mellitus, has been observed in about one-third of the patients.^1,3^ Herein, we report a case of a middle-aged male with CD that presented in our rheumatology department complaining of musculoskeletal pain, in whom metabolic bone disease was also found.

## CASE PRESENTATION

**Figure 1.**
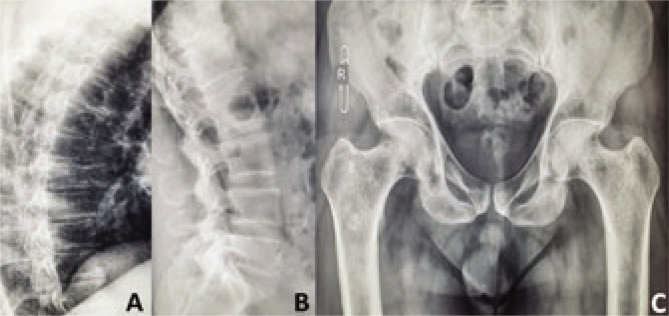
Plain radiographs of thoracic (A), lumbar (B) spine and pelvis-hips (C), showing diffuse osteopenia.

**Figure 2.**
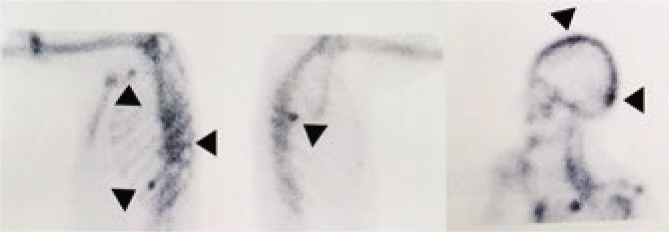
Bone scintigraphy depicting increased intake of Tc-99m in the skull, spine, anterior edge of the left 8th pleura, posterior edge of the right 5th pleura and the middle of the left clavicle (arrowheads), due to severe osteomalacia.

A 66-year-old man, presented with arthralgias in hips and knees for several months and accompanying chronic fatigue. He did not report any past personal or family medical history. Physical examination findings were unremarkable, except from mild knee osteoarthritis, which was attributed to his occupation as a farmer. An extensive work-up, including whole-body computed tomography and laboratory tests, ruled out malignancies, hematologic and rheumatic disorders. However, serum calcium and phosphorus levels were found at the lower normal limits, along with high parathormone (PTH) and alkaline phosphatase (ALP) (**Table 1**). Iron deficiency anaemia and severe deficiency of 25(OH)vitamin D_3_ (5.2 ng/mL) were also revealed. Plain X-rays exhibited diffuse osteopenic appearance of spine and hips (**Figure 1**). Bone scintigraphy was performed due to increased ALP and showed diffuse intake in the skull and the spine (**Figure 2**). In addition, focal high isotope intake was evident at the anterior edge of the left 8th pleura, the posterior edge of the right 5th pleura and the middle of the left clavicle, indicating possible incomplete fractures at these points (**Figure 2**). Bone mineral density (BMD) was severely reduced (T-score in left femoral neck −2.9 SD and −5.7 SD in L3-L4 vertebras), as shown in dual-energy X-ray absorptiometry (DXA). Laboratory, scintigraphic, and DXA findings were indicative of osteomalacia. Notably, patient recalled having chronic diarrhoea for several years, which he considered not important and compatible with his vegetable-rich nutrition. Upper gastrointestinal tract endoscopy demonstrated a non-specific gastroduodenitis and flattening of duodenal fold. Intestinal biopsy revealed intraepithelial lymphocytes, cryptic hypertrophy and moderate to severe villous atrophy, findings compatible with CD. A following screening for CD-specific auto-antibodies showed significantly elevated levels of IgA antibodies against tissue transglutaminase (anti-tTg), establishing the diagnosis of CD.

**Table 1.** Laboratory work-up of the patient.

**Parameter**	**Normal Range**	**Baseline**	**After 6 months of GFD treatment**
Ht (%)	38–52	**34.2**	41.4
Hb (g/dL)	13–18	**10.5**	14.2
MCV/MCH	79–100/26–32	82/**25.4**	89.2/30.6
CRP (mg/dL)	<0.5	**0.6**	0.1
ESR (mm/h)	<28	**34**	**34**
RF/anti-CCP/ANA	Negative	Negative	Negative
Fe (mg/dL)	65–175	**19**	143
Ferritin (ng/mL)	21–274	**7.4**	68.9
B12 (pg/mL)	179–1162	210	**1300**
25-OH-VitD3 (ng/mL)	<20	**5.2**	26.2
Serum phosphorus (mg/dL)	2.5–4.5	2.7	3.4
Serum calcium (mg/dL)	8.4–10.2	8.5	9.5
PTH (pg/mL)	11–62	**315.5**	23.2
ALP (IU/L)	40–140	**335**	91
anti-tTG IgA (RU/mL)	<20	**>200**	N/A

Values in bold are out of normal limits.

GFD: gluten-free diet; Ht: haematocrit; Hb: haemoglobin; MCV: mean corpuscular volume; MCH: mean corpuscular haemoglobin; CRP: C-reactive protein, ESR: erythrocyte segmentation rate; RF: rheumatoid factor; anti-CCP: antibodies against cyclic citrullinated peptide; ANA: anti-nuclear antibodies; PTH: parathormone; ALP: alkaline phosphatase; anti-tTG: antibodies against tissue transglutaminase

Gluten-free diet (GFD) and supplementation with calcium (1000 IU daily), and cholecalciferol (25000 IU twice a week for the first month, following 25.000 IU weekly for the next five months), were introduced. Six months later, patient reported amelioration of fatigue, arthralgias, myalgias and diarrhoea. In addition, haemoglobin, ferritin, calcium, phosphorus, 25(OH)-vitamin D3, PTH and ALP levels normalised (**Table 1**). An impressive 16% improvement of BMD in both lumbar spine (T-score −4.0 SD in L3-L4 vertebras) and hip (T-score −1.6 SD in the left femoral neck) was also observed. After 10 months of follow-up, the patient continues the GFD, along with calcium and cholecalciferol supplements, while he will soon start treatment with bisphosphonates.

## LITERATURE REVIEW

### Methods

An extensive review of the existing English-language literature was conducted using the PubMed database. The main terms used for database search were “osteomalacia”, “osteomalacia” OR “osteoporosis” OR “metabolic bone disease” AND “celiac disease”, “secondary osteoporosis”, “musculoskeletal manifestations” OR “arthritis” OR “arthralgia” AND “celiac disease”. The applied literature search focused, but not confined, to the most recent articles and last update was in June 2022.

### Clinical manifestations of CD

Clinical picture, serological tests and duodenal biopsy are the basis for the diagnosis of CD. Clinical presentation of patients with CD varies significantly. About 21% are asymptomatic at diagnosis, 27% present with the classical picture of diarrhoea, weight loss or malabsorption, whereas in 52% other manifestations are evident, such as constipation, anaemia, osteoporosis, elevated liver function tests, neurologic disorders, or dermatitis herpetiformis.^1^ Moreover, according to Yanming Xing et al., 50% of CD cases were diagnosed randomly in the screening for anaemia, low bone mass, transaminasemia, or infertility.^4^ It is, therefore, obvious that CD can be easily misdiagnosed.

### Musculoskeletal manifestations of CD

Osteoarticular manifestations like unexplained joint or muscle pain are not rare in CD. Jericho et al demonstrated that an important number of CD patients presented with arthralgia (16%), arthritis (15%) or myalgia (8%) at the time of diagnosis.^2^ A meta-analysis showed a cumulative incidence of arthralgia of 30.3% (19.2–42.8%) and bone pain of 6.1% (0.5–17.0%).^6^ However, the investigators did not find an increased risk for arthritis development compared to non-CD controls (OR=0.76 [95% CI: 0.16–3.66]).^6^ As for back pain, 45.2% of CD patients reported this symptom in a questionnaire-based cohort study.^7^ In some cases, sacroiliitis or enthesitis can be evident in imaging.^8^ In an earlier study with 200 CD patients, three forms of arthritis could be recognized: asymmetric oligoarthritis (often self-limiting and non-erosive), axial disease (tenderness over the spine and mild sacroiliitis), or both.^9^ To be noted, CD with arthritis exhibited elevated inflammation markers, compared to those without arthritis.^9^ On the other hand, about 2.4% of patients with unexplained articular manifestations, might have CD.^10^ Interestingly, these patients do not seem to respond to corticosteroids.^10^ Fortunately, arthralgias, arthritis and myalgias respond successfully in about 54%, 69% and 50% of the patients, respectively, after 2 years of GFD therapy.^2^ The positive impact of GFD on these symptoms seems to be even better in children with CD^2^; however, the response rate of articular symptoms to GFD is probably lower compared to gastrointestinal symptoms.^7^

### Metabolic Bone Disease in CD – Pathogenesis and Epidemiology

The pathogenesis of CD is currently not fully understood. Nevertheless, it is known that exposure to gluten triggers a pro-inflammatory innate immune response, assisted by a Th1/Th2 imbalance, that leads to villous blunting, increased intraepithelial lymphocytes infiltrates and subsequent intestinal damage.^1,11^ Intestinal inflammation, production of pro-inflammatory cytokines, and the presence of neutralising antibodies against osteoprotogerin (OPG) enhance bone absorption, contributing to the decreased BMD that is noted in these patients.^12,13^ One study exhibited a higher percentage of anti-OPG antibodies in CD patients (10%), compared to the general population.^14^ It has been also reported that OPG/RANKL ratio is lower in CD patients, compared to controls, and is positively correlated with spine BMD.^15^ Hormones imbalance that characterizes women with CD might possibly contribute in osteoporosis pathogenesis.^3^

On this basis, it is not surprising that low BMD can be found in up to 75% of CD patients regardless of intestinal disease activity.^3^ Patients with CD carry a 2.7-fold risk for osteoporosis development, compared to age- and sex-matched healthy controls.^6^ In a study of 563 premenopausal women and men diagnosed with CD, osteoporosis and osteopenia prevalence was 14.4% and 39.6%, respectively.^16^ Regarding specific sites, osteoporosis in the lumbar spine has been reported in 15–38% of the patients and in the hip in 18–44%.^12^ Importantly, 1.6% of patients with osteoporosis might have biopsy-proven CD.^17^ Furthermore, CD patients face a 7-fold increased fracture risk, compared to age- and sex-matched general population.^12^ In this line, a study from Sweden showed a relative risk of 2.1 (95% CI, 1.8–2.4) for hip fractures and 1.4 (95% CI, 1.3–1.5) for any fracture in 13,000 CD patients, compared to 65,000 age- and sex-matched controls, in a general population-based cohort.^18^

In patients with CD, excessive vitamin D and calcium malabsorption, due to villous atrophy, leads to secondary hyperparathyroidism, dramatically reduced bone mineralisation and osteomalacia.^11,12^ One meta-analysis showed a cumulative osteomalacia incidence of 18.3% in CD patients.^6^

### Metabolic Bone Disease in CD – Management

There are several recommendations regarding metabolic bone disease management and DXA examination in CD patients. The treatment of low BMD in CD mainly consists of GFD initiation and calcium/vitamin D supplementation.^6^ British Society of Gastroenterology and American College of Gastroenterology suggest to measure serum calcium, ALP, 25(OH)-vitamin D3 and PTH at the time of CD diagnosis, as it is essential to correct mineral metabolism abnormalities, such as hypocalcaemia, vitamin D deficiency and secondary hyperparathyroidism.^19,20^ In addition, DXA is recommended at the time of diagnosis and/or after one year on GFD in individuals over 55 years or among individuals with two or more risk factors for osteoporosis.^19–21^

Such risk factors include prior fragility fracture, corticosteroid use, physical inactivity, anticonvulsant use, smoking, alcohol excess, family history of osteoporotic fracture, female sex, early untreated menopause (<45 years), late menarche (>15 years), low daily calcium intake, age over 70 years, weight loss >10%, BMI <20 kg/m^2^, or poor correspondence to GFD after 1 year.^21^

The introduction of GFD improves BMD early, even within the first 12 months, but further improvement has been also reported later in the course of the therapy.^11^ However, in adult CD population, compared to children with CD, normalisation of BMD is less feasible.^11^ Thus, it is crucial to encourage these patients to follow the general non-pharmacological measures for osteoporosis; smoking cessation, limitation of alcohol intake and weight-bearing exercises are of great importance. Only few data are available for the effect of pharmacological treatment on osteoporosis of CD patients.^22^

Postmenopausal women and men aged over 50 years with osteoporosis not ameliorated with GFD, those with a fragility fracture or those at high risk for osteoporotic fracture are candidates for anti-osteoporotic therapy.^11,15,22^ Subsequently, it is recommended to repeat DXA in 1–2 years in people with low BMD or with no satisfactory response to GFD.^1,11,19–21^

## DISCUSSION

CD is a multisystemic autoimmune disorder with a wide spectrum of clinical manifestations, including musculo-skeletal symptoms.^8^ Importantly, in some cases arthralgia and/or myalgia might be the presenting symptoms, even in the absence of gastrointestinal complaints.^8^ We presented a case of a middle-aged CD patient that referred to a rheumatology clinic complaining of articular and back pain. It is known that up to 30% of CD patients might experience arthralgia or bone pain,^6^ while back pain might be even more common.^7,8^ However, data regarding arthritis development remain controversial.^6,8^

In our case, joint and back pain was not accompanied by synovitis or sacroiliitis. Regarding articular symptoms management, pain is gradually diminishing after GFD implementation.^2^ Indeed, GFD and vitamin D/calcium supplements were sufficient to ameliorate joint and muscle pains after only 6 months in our patient. To be mentioned, the latest guidelines support the measurement of disease-specific autoantibodies 12 months after GFD initiation to check for patient’s good adherence to GFD.^23^ We did not re-evaluate anti-tTG titres yet, firstly because of the shorter follow-up time and secondly due to the evident clinical and densitometric improvement.

Significantly reduced BMD was shown in our patient, and this concealed an underlying osteoporosis or osteomalacia. Although both disorders can coexist, scintigraphic findings and severe vitamin D deficiency were suggestive of osteomalacia in our case. It is a fact that osteomalacia may be confused with osteoporosis, as BMD is found decreased in both conditions. Osteoporosis is characterised by an imbalance between bone formation and bone absorption, leading in disturbed microarchitecture of a normally mineralized bone, while osteomalacia is a condition of reduced bone mineralization. Bone mineral:matrix ratio is normal (or slightly increased) in osteoporosis, whereas in osteomalacia is low.^24^ Osteoporosis is asymptomatic unless a fracture occurs; in contrast, osteomalacia can present with chronic fatigue, bone and joint pain, and proximal weakness in the late stage. Moreover, bone scintigraphy is useful for the differential diagnosis. In osteomalacia there is usually an increased tracer uptake throughout the skeleton, which sometimes can be so generalized and is characterised as “super-scan”.^25,26^ In some cases, focal increased uptake might unmask pseudofractures^25,26^ Diffuse uptake in skull and spine and pseudofractures in ribs and left clavicle were observed in our patient. In contrast, low tracer uptake is noted in osteoporosis, which might exhibit a ‘washed out’ pattern.^27^ Lastly, laboratory abnormalities, such as low vitamin D, serum calcium, phosphorus, and elevated PTH and ALP, are prominent in osteomalacia, in contrast to osteoporosis. Beyond osteoporosis-osteomalacia differential diagnosis, given that CD affects mainly premenopausal women and young men,^3^ CD should be ruled out in low-risk individuals with decreased BMD.

In conclusion, CD is associated with a wide range of musculoskeletal manifestations, such as arthralgia, arthritis, back pain, myalgia, osteomalacia, osteopenia and osteoporosis. As patients might refer to a rheumatologist for these complains, increased awareness is needed to ensure an earlier diagnosis of CD. In this line, in cases of unexplained joint or muscle pain, arthritis or low BMD, serological testing for CD is a reasonable option. GFD is *sine qua non* for these patients and successful adherence, accompanied by supplementary pharmacologic treatment, leads to elimination of minerals’ malabsorption, BMD improvement, fracture prevention and amelioration of joint symptoms.
